# PET/CT with [^68^Ga]gallium-oxine-labeled heat-denatured red blood cells for detection of dystopic splenic tissue

**DOI:** 10.1007/s00259-020-04899-4

**Published:** 2020-06-15

**Authors:** Martin Freesmeyer, Sebastian Gröber, Julia Greiser, Philipp Seifert, Falk Gühne, Robert Drescher

**Affiliations:** Clinic of Nuclear Medicine, Jena University Hospital, Am Klinikum 1, 07747 Jena, Germany

## Image of the month

Differentiation of harmless splenic tissue from malignancies and metastases can be challenging [1]. Accuracy of scintigraphy with [^99m^Tc]technetium-marked heat-denatured red blood cells (hd-RBC) is limited by low spatial and temporal resolution. Tracers for positron emission imaging of RBCs and platelets were proposed in 1977 but not developed further [2]. In 2017/2018, [^18^F]fluoride-labeled RBCs were successfully evaluated in animal studies [3, 4].

We report the first-in-human use of [^68^Ga]gallium-oxine-labeled hd-RBC for detection of dystopic splenic tissue. The PET tracer was validated according to Good Manufacturing Practice (GMP) guidelines [5]. Erythrocytes separated from 10 ml blood were labeled with 500 MBq [^68^Ga]Ga-oxine synthesized from [^68^Ga]Ga-chloride and 8-hydroxyquinoline (oxine) at 48 °C for 10 min, simultaneously achieving heat denaturation of the blood cells. Labeling efficiency was > 95%.

In a 73-year-old man with a history of renal cell carcinoma (RCC), PSMA-PET/CT showed three suspicious lesions: two pulmonary nodules, which were resected and histologically proven to be RCC metastases, and a perisplenic lesion. Hd-RBC scintigraphy performed for this lesion showed low, indistinct uptake and could not clearly differentiate an accessory spleen from a metastasis (a, arrows).

PET/CT with 150 MBq [^68^Ga]Ga-oxine-hd-RBC showed intense tracer uptake in the spleen and the perisplenic nodule (b, arrows), identifying it as splenic tissue. High vascular contrast was evident (b, c). One minute after injection of [^68^Ga]Ga-oxine-labeled hd-RBC, tracer distribution in the spleen is analogue to organ perfusion (d, arrow). Three minutes p.i., splenic uptake was equal to activity in the blood. Five minutes p.i., it markedly exceeded blood pool activity, suggesting erythrocyte sequestration.




